# Minimum clinically important differences in the Minnesota Living with Heart Failure questionnaire: from a study of heart failure patients treated with integrated Chinese and Western medicine

**DOI:** 10.3389/fcvm.2023.1242216

**Published:** 2023-11-27

**Authors:** Yanbo Zhu, Jianni Cong, Lin Lin, Jinhang Du, Liqun Long, Yuan He, Jiaju Ren

**Affiliations:** ^1^ School of Management, Beijing University of Chinese Medicine, Beijing, China; ^2^ School of Chinese Medicine, Beijing University of Chinese Medicine, Beijing, China; ^3^ Department of Personnel, China-Japan Friendship Hospital, Beijing, China; ^4^ Cardiology Department of Integrated Traditional Chinese and Western Medicine, China-Japan Friendship Hospital, Beijing, China; ^5^ School of Computer Science and Statistics, Trinity College, Dublin, Ireland

**Keywords:** Minnesota Living with Heart Failure questionnaire, minimum clinically important difference, heart failure, quality of life, integrated Chinese and Western medicine

## Abstract

**Objective:**

The purpose of this study was to estimate the minimum clinically important differences (MCIDs) in the Minnesota Living with Heart Failure questionnaire (MLHFQ), which targeted patients with heart failure treated with integrated Chinese and Western medicine, as a means of helping doctors and patients judge the effectiveness of intervention.

**Methods:**

A total of 194 patients with chronic heart failure were recruited from three general hospitals in Beijing. Anchor-based and distribution-based approaches were used to estimate MCID. The anchor was SF-36 item 2 (HT, Health Transition), and the calculation methods included the mean change method, receiver operating characteristic (ROC) curve analysis, and linear regression model. For the distribution-based approaches, 0.2, 0.5, and 0.8 standardized response mean (SRM) values and standard error of measurement (SEM) value of 1 were used.

**Results:**

The correlation coefficients of the MLHFQ scale information and HT were 0.346–0.583. Different MCIDs were obtained by the mean change method, ROC curve, and linear regression model. The minimum MCID in the physical domain, emotional domain, and total scores were 3.6, 2.0, and 7.4, respectively; the maximum estimates were 9.5, 2.5, and 13.0, respectively; and the average estimates were 5.7, 2.2, and 10.0, respectively. The average estimates were close to the result of the 0.5 SRM or 1 SEM.

**Conclusion:**

We established MCIDs in the MLHFQ using anchor-based and distribution-based approaches. It was recommended to round the average estimates of anchor-based approaches up to the nearest whole number for the MCIDs of the MLHFQ physical domain, emotional domain, and total scores. The results were 6.0, 2.0, and 10.0, respectively.

## 1. Introduction

Heart failure (HF) is common worldwide, affecting at least 26 million patients globally (1). In China, HF affects more than 13 million people and represents the second largest cause of death (2, 3). With the rapid increase in population age, the prevalence and mortality of HF will continue to increase, imposing significant social burdens. HF is a progressive, ultimately fatal, long-term disease. Although existing treatments can delay, they generally do not reverse the progression of HF (4). In recent years, comprehensive therapy for HF has reduced the overall mortality and re-hospitalization rates. However, the physical symptoms and psychological pain associated with HF often leads to poor quality of life for patients (5).

In the early 1990s, the primary outcomes used in the assessment of HF treatment effectiveness were mortality and re-hospitalization rates (6). Because of the transformation of medical models and the appearance of positive health perspectives, the management of chronic diseases such as HF should not only be concerned with prolonging the life of patients but also focus on the improving the subjective health of patients, reducing the symptoms of the disease, and improving the subjective satisfaction of patients. This goal can be well achieved by combining quality-of-life assessments with traditional evaluation indicators (such as mortality) (7). Quality-of-life evaluations are attracting increased attention in clinical research, and they have been widely used in clinical effectiveness evaluations of chronic diseases such as HF, health service program evaluations, and in other fields (8, 9).

The Minnesota Living with Heart Failure questionnaire (MLHFQ) is a specific instrument commonly used in clinical studies to evaluate the quality of life of patients with HF (10). In clinical studies, researchers generally take whether the difference in MLHFQ score before and after intervention is statistically significant as the evaluation standard. However, a statistically significant difference related to the sample size can only indicate that a change was not coincidental and cannot be used to conclude that the intervention would bring substantial benefits to patients. Therefore, for doctors or patients, a statistically significant difference does not equal a clinically significant difference. The minimum clinically important difference (MCID) is the smallest difference in the scale dimension score that patients perceive as beneficial in the absence of side effects and excessive cost (11). The MCID can provide a critical threshold for evaluating the effectiveness of an intervention from the perspective of both physicians and patients and intuitively inform physicians and patients as to whether the intervention is effective. In other words, if the difference in the scale score reaches the MCID, the intervention is effective. Determination of the MCID can make the scale score more intuitive, and it can be easily used for clinical applications to measure the control of blood pressure, blood glucose, etc. To our knowledge, no MCIDs for the MLHFQ instrument have been determined in China. Thus, this study aimed to estimate the MCIDs of the MLHFQ using clinical data from a study of patients with chronic HF treated by integrated Chinese and Western medicine. The objective was to provide a basis for judging the effectiveness of the intervention for physicians and patients to facilitate targeted management of cardiovascular diseases such as HF.

## 2. Methods

### 2.1. Study participants

This study was a secondary study using data collected by the National Natural Science Foundation of China (NSFC) project (no. 30873256). The details of the NSFC project design were reported elsewhere (12). A total of 199 hospitalized patients with chronic HF were recruited from inpatient departments of three general hospitals in Beijing between September 2009 and December 2011. All eligible patients were treated by syndrome differentiation and Chinese medicine based on the conventional therapy for 2 weeks. Surveys were conducted before intervention and 2 weeks after intervention. Information for four patients was incomplete, and 1 patient was lost to follow-up during the second investigation. Finally, 194 patients were included in the analysis. Our study did not carry out additional interventions and only assessed clinicians’ actual treatment. All participants signed informed consent forms and voluntarily participated. All patients satisfied New York Heart Association (NYHA) criteria I–IV. Patients with mental illness, congenital heart disease, or severe impairment of liver or kidney function were excluded. Pregnant patients, patients with allergies, and patients with acute myocardial infarction or unstable angina pectoris within the last month were also excluded.

### 2.2. Instruments

The MLHFQ was used to evaluate the quality of life of the enrolled patients. The MLHFQ is a commonly used instrument for HF patients to self-assess their quality of life, and it is widely used globally (13–15). The MLHFQ contains 21 items scored on a six-point Likert scale ranging from 0 to 5. Total scores ranged from 0 to 105, representing the best to worst quality of life. The MLHFQ has two domains, a physical domain (eight items, score range 0–40) and an emotional domain (five items, score range 0–25), with the remaining 8 items only used to calculate the total score (16). Yanbo et al. (17) got the MLHFQ copyright license and developed a Chinese version of the MLHFQ that has been verified in China.

### 2.3. Anchor

Item 2 (HT, Health Transition) of Short Form-36 (SF-36) was used as the anchor in the current study. The SF-36 instrument focuses on generic quality of life (18); the HT of SF-36 is a single item measuring health changes and commonly used as an anchor in anchor-based approaches (19). In our study, the survey time of HT was adjusted to compare with the pre-admission period (Compared to pre-admission ago, how would you rate your health in general now: (1) Much better; (2) Somewhat better; (3) About the same; (4) Somewhat worse; (5) Much worse). Those who chose option 1 showed significant improvement in health, whereas those who chose option 2 showed slight improvement in health, those who chose option 3 showed no improvement, and those who chose option 4 or 5 showed deterioration in health.

### 2.4. Statistical analysis

The Shapiro–Wilk test was used to analyze the normal distribution of continuous variables, and the nonparametric Wilcoxon signed rank test was used to analyze differences in the various domain scores of the MLHFQ before and after intervention. Spearman correlation analysis was conducted to analyze the correlations between anchor (HT) and MLHFQ information, and the correlation threshold for the anchor and scale information was 0.3–0.35, as recommended by Revicki et al. (20). MCIDs were calculated using common anchor-based and distribution-based approaches (21).

Three statistical algorithms were used for the anchor-based approaches. The first was the mean change method, which is most frequently reported in the literature (22). The MCIDs are determined according to the changes in scores of the slight improvement group before and after an intervention in longitudinal studies. If the change in scores is normally distributed, the mean of the change in scores from the slight improvement group is the MCID. If the change in scores is non-normally distributed, the MCID is the median of the change in scores from the slight improvement group. The second method is the receiver operating characteristic (ROC) curve. In this study, non-parametric ROC curves were used to determine MCIDs, and the gold standard was HT classification, with the MLHFQ representing the new standard to be tested. The area under the ROC curve (AUC) represents the probability of correctly distinguishing between patients with improved health status and patients without improved health status. The AUC ranges from 0.5 to 1.0, representing an undifferentiated discrimination (50% sensitivity and 50% specificity) and perfect discrimination (100% sensitivity and 100% specificity), respectively. Terwee et al. recommended that the AUC should not be lower than 0.7 (23). As the AUC becomes closer to 1, the accuracy of the judgment increases. The MCID is the value of the point closest to the upper left corner of the ROC curve; that is, it is the estimate of the point with the highest Youden index (sensitivity + specificity−1). The third method is linear regression model, which can include all patients and control the influences factors such as gender and age on the dependent variables. The difference score of MLHFQ before and after intervention in various domains was taken as the dependent variable and HT as the independent variable to establish a binary linear regression model, covariables such as gender and age could also be included to establish a multiple linear regression model, and the slope of the regression model was MCID (24).

Distribution-based approaches are used to estimate MCIDs from a statistical perspective based on the distribution of sample data. In our study, the standardized response mean (SRM) and standard error of measurement (SEM) were used to estimate MCIDs (25).

$$\mathrm{MCID}\;=\;\mathrm{SR}M^{*}SD_{\mathrm{diff}}\;\mathrm{or}\;\mathrm{MCID}=\mathrm{SE}M^{*}SD_{0}{}^{*}\sqrt{1-r},$$

where SD_diff_ represents the standard deviation of MLHFQ scores before and after the intervention, SD_0_ represents the standard deviation of MLHFQ scores before the intervention, and *r* represents the reliability coefficient of the measurement instrument. For the measurement outcome of the scale, *r* generally indicates test-retest reliability, Cronbach's α can be used instead for test-retest reliability, and 1 SEM can be calculated as the estimated value of the MCID (26). MCID values were calculated at SRM values of 0.2, 0.5, and 0.8, indicating low, medium, and high effects, respectively (25, 27).

All statistical analyses were performed using SPSS 23.0 software.

## 3. Results

### 3.1. Patient characteristics

In the current analysis, 194 eligible patients were included, and the patient characteristics are shown in Table 1. The proportion of male and female subjects was the same, with average age 69.48(11.69) years, the youngest being 27 years old and the oldest 88 years old. More than half of the patients (57.73%) had an educational level of middle school, high school, or technical secondary school, and the NYHA scale was mainly grade II (26.29%) and grade III (54.12%) before intervention.

**Table 1:** Patient characteristics.

Parameter	Classification	*n*	Proportion (%)
Gender	Men	97	50.00
	Women	97	50.00
Age (years)	<60	40	20.62
	60–69	35	18.04
	70–79	85	43.81
	≥80	34	17.53
Education	Primary school and below	50	25.77
	Junior high school, high school, and technical secondary school	112	57.73
	College degree and above	32	16.49
Smoking history	Yes	26	13.40
	No	128	65.98
	Quit	40	20.62
Alcohol history	Yes	21	10.82
	No	156	80.41
	Quit	17	8.76
Family heart diseases history (*n* = 192,2 cases missing)	Yes	37	19.27
	No	155	80.73
HF course (*n* = 188,6 cases missing)	<1 year	46	24.47
	1–4 year	68	36.17
	5–9 year	32	17.02
	≥10 year	42	22.34
Comorbidity	Coronary heart disease (*n* = 190, 4 cases missing)	93	48.95
	Hypertension (*n* = 191, 3 cases missing)	121	63.35
	Diabetes (*n* = 187, 7 cases missing)	63	33.69
	Cerebrovascular disease (*n* = 189, 5 cases missing)	14	7.41
	COPD, Asthma, Arthritis, etc. (*n* = 185, 9 cases missing)	11	5.95
NYHA	I	2	1.03
	II	51	26.29
	III	105	54.12
	IV	36	18.56

### 3.2. MLHFQ outcomes before and after intervention

Table 2 shows that after intervention, the total score, physical domain score, and emotional domain score of the MLHFQ were significantly lower than before the intervention (*P *< 0.01). The correlations between the changes in MLHFQ scores and NYHA before and after intervention were 0.083–0.184, whereas those between MLHFQ scores and HT were 0.346–0.583.

**Table 2:** MLHFQ outcomes before and after intervention.

	Physical domain	Emotion domain	Total score
Before intervention	23.95 ± 12.18	9.90 ± 6.95	50.13 ± 26.47
After intervention	15.10 ± 10.03	6.43 ± 5.30	33.27 ± 21.51
*d*	8.85 ± 8.59	3.47 ± 5.14	16.91 ± 17.58
z	10.580	8.465	10.489
*P*	<0.001	<0.001	<0.001

### 3.3. MCIDs of the MLHFQ

#### 3.3.1. Anchor-based approaches

##### 3.3.1.1. Mean change method

With HT as the anchor, 79 patients showed slight improvement in health status, 84 patients showed significant improvement, 24 patients showed no improvement, and seven patients showed deterioration in health status. Supplementary Table S1 showed the comparison of MLHFQ scores between the four groups before and after intervention. MLHFQ scores of intervention before were higher in the significantly improved group (*P* < 0.05).The changes in the scores of the MLHFQ in various domains were not normally distributed before and after intervention, as determined by Shapiro–Wilk analyses (Supplementary Table S2). Therefore, the median changes in the scores of the slight improvement group represented the MCIDs of the MLHFQ. The values were 6.0, 2.0, and 13.0 for the physical domain, emotional domain, and total score, respectively (Table 3).

**Table 3:** MCIDs of the MLHFQ based on the mean change method.

MLHFQ	Median			
	No improvement	Slight improvement	Significant improvement	MCID
Physical domain	2.0	6.0	11.5	6.0
Emotion domain	0.0	2.0	6.0	2.0
Total score	3.5	13.0	24.0	13.0

##### 3.3.1.2. ROC curve

**Figure 1:**
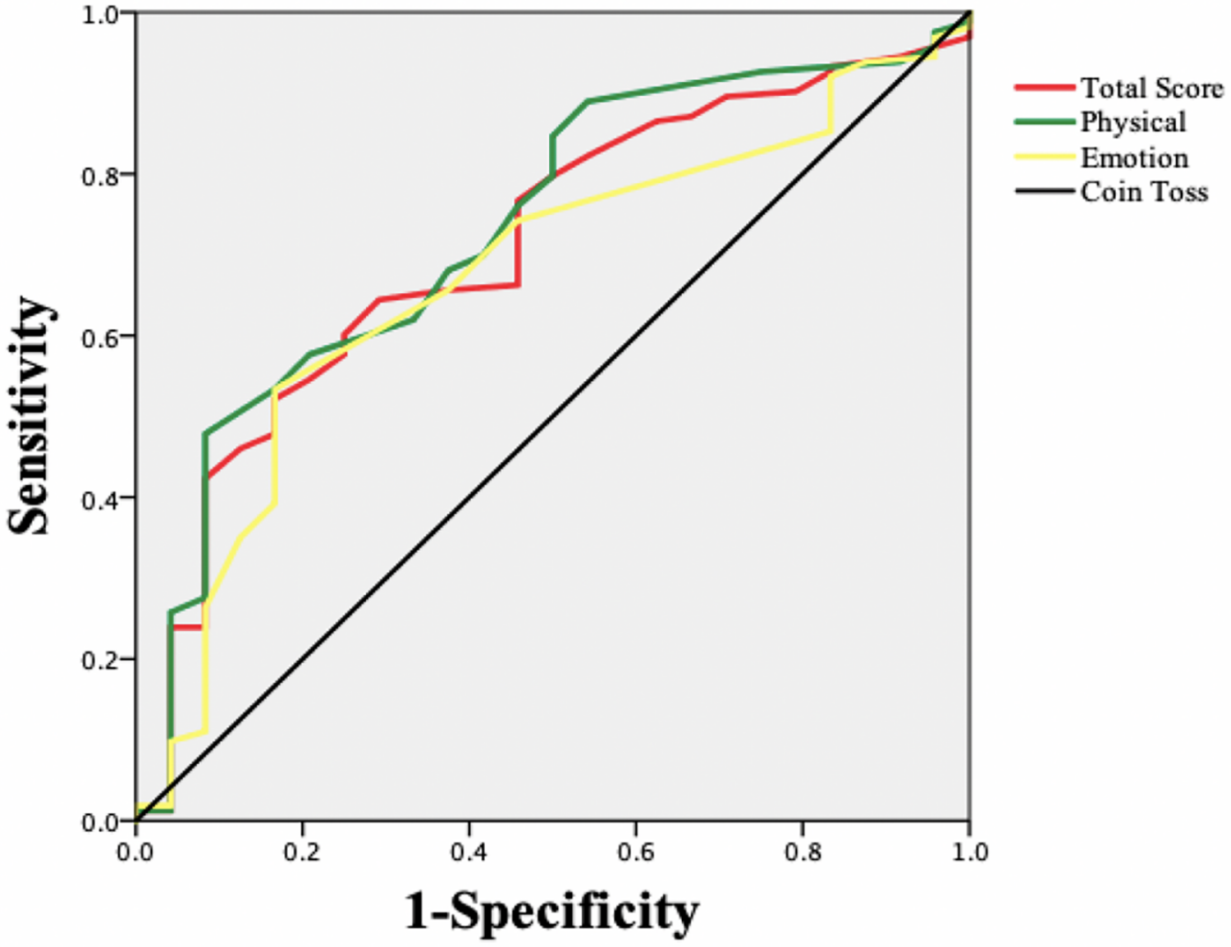
ROC curves for the MLHFQ based on an HT anchor.

An ROC curve was constructed taking the HT classification as the “gold standard” and the MLHFQ as the new standard for evaluation (Figure 1). Improvement in health status was defined as positive, and no improvement in health status was defined as negative. Table 4 shows that the areas under the ROC curves for each MLHFQ domain constructed based on the HT anchor ranged from 0.673 to 0.732, and the MCIDs of MLHFQ physical domain, emotional domain, and total score were 9.5, 2.5, and 12.0, respectively.

**Table 4:** MCIDs of the MLHFQ based on ROC curves.

	AUC	95% CI	Sensitivity	Specificity	MCID
Physical domain	0.732	0.627–0.836	0.479	0.917	9.5
Emotion domain	0.673	0.563–0.782	0.534	0.833	2.5
Total score	0.710	0.605–0.815	0.644	0.708	12.0

##### 3.3.1.3. Linear regression model

Table 5 showed that with HT as the independent variable, the MCIDs of the MLHFQ physical domain, emotional domain and total score were approximately 3.7, 2.2 and 7.7. Controlling gender, age, and education level, the MCIDs of the MLHFQ physical domain, emotional domain and total scores were approximately 3.6, 2.2, and 7.4. We also checked the non-linear regression model between the HT and the change scores in different domains of MLHFQ. The regression model ΔMLHFQ = a*exp(b*HT) was used to derive MCIDs. Supplementary Table S3 showed the MCIDs derived from the instantaneous slope of the curve.MCIDs derived from line regression model were slightly higher than that of non-linear regression model. Linear regression is a commonly used method for estimating slopes to calculate MCIDs in the literatures. The MCIDs calculated from the linear regression model were adopted as a conservative estimate of a meaningful change.

**Table 5:** MCIDs of MLHFQ based on linear regression model.

Dependent variable	MCID		
	Physical domain	Emotion domain	Total score
HT	3.692	2.192	7.708
HT, gender, age, education	3.564	2.152	7.448

##### 3.3.1.4. Comparison of MCIDs estimated by anchor-based approaches

The minimum, maximum, and average values of the anchor-based approaches determined by the mean change method, ROC curve analysis, and linear regression model were adopted to estimate the MCIDs of various domains in the MLHFQ, and the results are shown in Table 6.

**Table 6:** MCIDs of the MLHFQ determined using anchor-based approaches.

	Minimum value	Average value	Maximum value
Physical domain	3.6	5.7	9.5
Emotional domain	2.0	2.2	2.5
Total score	7.4	10.0	13.0

#### 3.3.2. Distribution-based approaches

The SRM and SEM were also used to estimate the MCIDs of the MLHFQ. Table 7 showed that the MCIDs when SEM were 1and SRM were 0.2, 0.5 and 0.8, respectively.The estimated value obtained at 1 SEM was close to the estimated value when the SRM was 0.5.

**Table 7:** MCIDs of the MLHFQ determined using distribution-based approaches.

	SD_0_	SD_diff_	MCID			
			0.2 SRM	0.5 SRM	0.8 SRM	SEM
Physical domain	12.18	8.59	1.72	4.30	6.87	5.31
Emotional domain	6.95	5.14	1.03	2.57	4.11	2.87
Total score	26.47	17.58	3.52	8.79	14.06	9.17

## 4. Discussion

The MCID was first proposed by Guyatt et al. in 1987 (28) and has received increasing attention in clinical practice since the initial report (29). The MCID is a patient-centered concept and reflects both the magnitude of disease improvement and the importance of change to patients. In clinical effectiveness evaluations, the MCID must be taken into account when evaluating the results of improvements in subjective states (30). The methods for estimating MCIDs mainly include anchor-based, ﻿distribution-based, expert consensus, and literature-based approaches. At present, there is no consensus regarding the best method for estimating MCIDs. Scholars generally recommend estimation strategies based on anchor-based approaches assisted by other methods (25). Anchor-based approaches and distribution-based approaches, which are the most widely reported in the literature, were adopted in the current study to determine the MCIDs of the MLHFQ for chronic HF patients treated with integrated Chinese and Western medicine. The determination of MCIDs for the MLHFQ could help physicians and patients better understand the treatment effects, weigh the pros and cons of treatments, and adjust targeted interventions. MCIDs can also be used as the basis for clinical trials to judge curative effects.

The estimation of MCIDs based on anchor-based approaches is affected by the selection of anchors or statistical methods, and MCIDs may differ if different anchors or statistical methods are used for their calculation. Anchors include objective and subjective types. Objective anchors are debated due their perceived disconnection to the concept of important or meaningful change (21).

In this study, the correlation between the changes in the scores of the MLHFQ and NYHA before and after intervention ranged from 0.083 to 0.184 but were lower than 0.3. These results indicated that the NYHA appears to be less sensitive to changes in scores of the MLHFQ and is therefore not suitable for use as an anchor. The HT is a subjective anchor based on a patient's perspective to evaluate their own improvement in health status and reflects a patient-centered concept (31). In this study, the correlation between MLHFQ scale information and HT before and after intervention ranged from 0.346–0.583. According to the threshold value of 0.3–0.35, the HT was acceptable as an anchor.

The results of this study showed some differences in the MCIDs of the MLHFQ in various domains as calculated using different statistical algorithms in anchor-based approaches. Scholars tend to recommend use of the mean change in score of the slight-improvement group to determine the MCID (22). MCIDs determined based on the mean change method can lead to as many as half of respondents being wrongly classified as having no response (ineffective intervention), so some scholars believe that the mean change method may not be an appropriate method for estimating the MCID at the individual level (21). However, this approach is still the most widely reported method in the literature. In this study, the MCIDs of the MLHFQ in the physical domain, emotion domain, and total score based on the HT were 6.0, 2.0, and 13.0, respectively.

Determining MCIDs based on ROC curve analysis can maximize the sensitivity and specificity and reduce wrong classifications. Therefore, some scholars believe that this approach is better than the mean change method and recommend the use of ROC curves to estimate MCID values at the individual level (32). However, other scholars believe that estimating MCIDs by ROC curve analysis is the best way to distinguish “slight improvement” from “no change” score differences. This approach identifies the best clinically important difference rather than the MCID (22). In this study, the AUC values of the physical domain and total score of the MLHFQ were 0.732 and 0.710, respectively, values that were both greater than 0.7. The AUC of the emotional domain was 0.673, slightly less than 0.7. The MCIDs of the physical domain, emotional domain, and total score were 9.5, 2.5, and 12, respectively. The sensitivity (true-positive rate) ranged from 0.479 to 0.644, and the specificity (true-negative rate) ranged from 0.708 to 0.917. As sensitivity was lower than the specificity, the MCIDs estimated by the ROC curve approach in our study were relatively high.

Linear regression model can make maximum use of data and control the influence of confounding factors. But it has high requirements on data. The data in the true word is difficult to fully meet the requirements. In our study, the MCIDs of physical domain, emotional domain and total score estimated by linear regression model were approximately 4.0, 2.0 and 8.0. The MCIDs were the lowest among the three statistical methods of anchor-based approaches and had higher sensitivity.

Each of the three above-mentioned statistical methods has its own specific advantages and disadvantages. In our study, the MCIDs of the MLHFQ were estimated by taking the minimum, average, and maximum values of the three methods. The MCIDs of the physical domain, emotional domain, and total score based on minimum values were 3.6, 2.0, and 7.4, respectively. The MCIDs determined using the maximum method were 9.5, 2.5, and 13.0, respectively, whereas those determined using the average method were 5.7, 2.2, and 10.0, respectively. Use of the minimum value can improve the sensitivity of the estimation, and use of the maximum value can improve the specificity. Based on the results of our study, the average value of the three statistical methods examined is recommended. For clinical application, it is suggested that the MCIDs of the MLHFQ physical domain, emotional domain, and total score be rounded up to the nearest whole number (e.g., 6.0, 2.0, and 10.0, respectively). The results were lower than the MCIDs of the MLHFQ from Spanish heart failure patient, whose results of physical domain, emotional domain, and total score were 9.17, 3.39, and 19.14 respectively (32). The differences may be related to cultural traditions, the baseline status of the patient, the choice of anchors, and so on.

Although distribution-based approaches are simple ways to estimate MCIDs, their use is debated by scholars because they relies solely on statistical inferences and do not reflect the clinical significance of the changes (33). Distribution-based approaches are therefore not ideal for estimating MCIDs, but their use as auxiliary methods can provide a threshold for interpreting MCIDs. Changes in scores that exceed MCIDs estimated using distribution-based approaches can be considered clinically significant (21). In our study, SRM and SEM were used in the distribution-based approaches, and the estimated MCID of 1 SEM was close to the estimated value at an SRM value of 0.5. The MCIDs of the MLHFQ in all domains estimated using anchor-based approaches were higher than the estimated values at SRM = 0.2 but close to the estimated values at 1 SEM or 0.5 SRM. Therefore, the MCIDs estimated using anchor-based approaches were reasonable. Moreover, some scales used in clinical research do not have a definite MCID and appropriate anchor. Thus, our results suggest that distribution-based approaches with SRM = 0.5 or 1 SEM should be considered in such cases for MCID determination instead of anchor-based approaches.

The generic, stable MCIDs can more effectively help clinicians evaluate the effect of intervention.MCID may be affected by various factors such as patients’ demographic characteristics, baseline status, and method used (34), but the MCID is a key threshold for assessing intervention from the perspective of the patient. It can guide the physician's clinical decision, and is a very attractive indicator in the clinic.Most the participants included in this study were over 60 years old and have certain basic diseases such as hypertension, coronary heart disease and so on. Their understanding of health may be different from that of young people and newly diagnosed patients, which may affect the MCIDs. This study found that baseline MLHFQ scores were higher in the significantly improved group, suggesting that those with more severe symptoms were more likely to change their scores after intervention,further confirming baseline status could affect MCIDs. MLHFQ has a floor effect and is not sensitive to identify the mild heart failure (35). Subsequent stratified studies based on patient health status at baseline may be required to further verify the stability of the results. In order to improve the accuracy of MCIDs as the threshold for evaluating intervention effects as much as possible, three methods of anchor-based methods were used, and the rationality of MCIDs estimated by the anchor-based method were further tested with the distribution-based approach. However, Considering the variability of MCIDs, MCID estimation may be affected not only by extrinsic factors such as study population and estimation method, etc., but also by intrinsic factors such as sample size and anchor etc. (36), the use of MCIDs alone may lead to incorrect classification of effectiveness assessment. It is recommended that clinicians need consider the characteristics of population and other outcome indicators comprehensively when using MCIDs.

As with all studies, our research has some limitations. First, there were few patients who exhibited deteriorating health status among the included subjects. Therefore, no MCIDs related to deteriorating health status were observed in our study. A study conducted in Spain showed that the MLHFQ is more responsive to health improvements than to worsening of health status and therefore does not adequately reflect changes in patients whose health deteriorates (32). Further verification of this issue is needed. Second, the NYHA classification of the inpatient subjects enrolled in this study was primarily grade II or III, and therefore, the estimated MCIDs may not be applicable to outpatients with mild symptoms. Third, the anchor HT was used mainly for evaluation of overall health changes in the patients. These changes may have been affected not only by the clinical interventions but also by factors such as the patients’ health expectations and family support, for example. In the future, the sample size should be expanded, and stratified studies should be conducted according to the scale baseline score, gender and other factors for different populations, to further verify the MCIDs estimated in our study.

## 5. Conclusions

In the present study, anchor-based and distribution-based approaches were used estimate MCIDs of the MLHFQ for patients treated with integrated Chinese and Western medicine. Based on our results, it is recommended that the average estimates of MCIDs of the MLHFQ physical domain, emotional domain, and total score determined using anchor-based approaches be rounded up to the nearest whole number. Using this approach, the results were 6.0, 2.0, and 10.0, respectively. These values represent the minimum change required for patients to perceive noticeable and beneficial accepted changes in their health following clinical interventions.

## Data Availability

The raw data supporting the conclusions of this article will be made available by the authors, without undue reservation.
