# Supplementary material for: Minimum clinically important differences in the Minnesota Living with Heart Failure questionnaire: from a study of heart failure patients treated with integrated Chinese and Western medicine
Source: Front Cardiovasc Med. 2023 Nov 27;10:1242216. doi: 10.3389/fcvm.2023.1242216 (PMC10711109; doi:10.3389/fcvm.2023.1242216)
Supplement: Supplementary file 1 [file Table1.docx]

**Minimum clinically important differences in the Minnesota Living with Heart Failure questionnaire:** **from** **a study of heart failure patients treated with integrated Chinese and Western medicine**

Supplemental data

Table S1 Comparison of MLHFQ scores before and after intervention

| MLHFQ |  | Deterioration^1^ | No improvement^2^ | Slight improvement^3^ | Significant improvement^4^ |
| --- | --- | --- | --- | --- | --- |
| Physical domain^a^ | Before intervention | 19.43±10.01 | 18.42±14.06 | 21.96±12.36 | 27.79±10.49 |
|  | After intervention | 10.71±5.99 | 14.63±13.03 | 15.85±10.85 | 14.90±8.45 |
|  | *z* | 2.375 | 2.580 | 6.082 | 7.753 |
|  | *P* | 0.018 | 0.010 | <0.001 | <0.001 |
| Emotion domain^b^ | Before intervention | 8.14±6.26 | 8.87±7.37 | 8.29±6.733 | 11.86±6.70 |
|  | After intervention | 6.86±5.79 | 7.38±6.90 | 6.51±5.963 | 6.05±3.982 |
|  | *z* | 1.604 | 1.316 | 4.226 | 7.008 |
|  | *P* | 0.109 | 0.188 | <0.001 | <0.001 |
| Total score^c^ | Before intervention | 33.86±17.51 | 40.75±30.56 | 44.84±26.26 | 59.15±23.30 |
|  | After intervention | 19.71±9.07 | 32.63±29.72 | 34.06±23.98 | 33.74±16.39 |
|  | *z* | 2.371 | 2.663 | 5.866 | 7.708 |
|  | *P* | 0.018 | 0.008 | <0.001 | <0.001 |

The comparison of scores (before intervention) among the four groups by LSD(Least significant Difference) test:

a:The differences of 2 and 4, 3 and 4 were statistically significant;

b:The differences of 3 and 4 was statistically significant.

c: The differences of 1 and 4, 2 and 4, 3 and 4 were statistically significant;

Table S2 The result of Shapiro–Wilk analyses about MLHFQ scores in various domains before and after intervention

| MLHFQ | *z* | *P* |
| --- | --- | --- |
| Physical domain | 2.181 | 0.015 |
| Emotion domain | 3.848 | <0.001 |
| Total score | 3.130 | <0.001 |

Table S3 MCIDs of the MLHFQ based on a non-linear regression model

| Optimum estimation value | Physical domain | Emotional domain | Total score |
| --- | --- | --- | --- |
| a | 3.539 | 0.685 | 5.881 |
| b | 0.639 | 1.066 | 0.725 |
| MCID | 3.166 | 1.304 | 6.262 |
